# Modularity in Protein Complex and Drug Interactions Reveals New Polypharmacological Properties

**DOI:** 10.1371/journal.pone.0030028

**Published:** 2012-01-18

**Authors:** Jose C. Nacher, Jean-Marc Schwartz

**Affiliations:** 1 Department of Complex and Intelligent Systems, Future University Hakodate, Hokkaido, Japan; 2 Faculty of Life Sciences, Manchester Interdisciplinary Biocentre, University of Manchester, Manchester, United Kingdom; Hospital for Sick Children, Canada

## Abstract

Recent studies have highlighted the importance of interconnectivity in a large range of molecular and human disease-related systems. Network medicine has emerged as a new paradigm to deal with complex diseases. Connections between protein complexes and key diseases have been suggested for decades. However, it was not until recently that protein complexes were identified and classified in sufficient amounts to carry out a large-scale analysis of the human protein complex system. We here present the first systematic and comprehensive set of relationships between protein complexes and associated drugs and analyzed their topological features. The network structure is characterized by a high modularity, both in the bipartite graph and in its projections, indicating that its topology is highly distinct from a random network and that it contains a rich and heterogeneous internal modular structure. To unravel the relationships between modules of protein complexes, drugs and diseases, we investigated in depth the origins of this modular structure in examples of particular diseases. This analysis unveils new associations between diseases and protein complexes and highlights the potential role of polypharmacological drugs, which target multiple cellular functions to combat complex diseases driven by gain-of-function mutations.

## Introduction

The complex interweave of interactions between proteins plays a key role for many cellular processes. Protein complexes emerge as temporally stable compounds to perform precise biological functions through the association of proteins by means of non-covalent protein-protein interactions. The fact that relatively similar genome sizes give rise to drastically different organism's complexity [1] has raised the question of what mechanisms constrain the huge number of possible protein complexes [2]–[4]. On the other hand, the information on protein-protein interactions and protein complex formation does not only provide a better understanding of molecular evolution [5], but it may also improve our knowledge of human disorders and lead to new strategies for therapeutic intervention.

Since the number of discovered single-target drug is not increasing as fast as could be expected based on our current knowledge of the genome, several novel approaches have been suggested such as the development of multi-target drugs. One of the drawbacks for the fast development of such drugs is that it is not easy to experimentally test the response of a complex system to a multi-target drug, unless in vivo experiments are performed. But beyond experimental constraints, several works have suggested that multiple but partial attacks on specific targets can be more efficient that the knockout of a single target [6]. In Ref. [7], several studies on human system-drug interactions were discussed. Drug-target networks have recently been extensively investigated [8]–[11]. Moreover, the human disease-gene network was reported in [12], and the interactions between therapy and drugs at different ATC levels studied in [13]. The regulation of drug targets by miRNA was also extensively analyzed [14]. But in spite of their importance, the intricate web of interactions defined by the human associations of protein complexes and all available drugs remains uncharacterized. This network represents a higher level view of the interactions between drugs and life molecules since each molecular complex is also composed of individual proteins as subunits. As shown by Ref. [7], networks with higher complexity could also be explored by considering drug – symptoms and drug – patients associations.

While previous works have focused on specific complexes that lead to human disorders, until recently protein complexes were not identified and classified in a sufficiently comprehensive way for a systematic analysis of the human protein complex system to be performed. We here present a large-scale analysis of the global set of relationships between all available drugs and human protein target complexes. By using the resulting network, it should be possible to elucidate to what extent complexes interact with drugs as well as to uncover specific links between diseases and protein complexes. Related approaches have dealt with the problem of providing a global, network-based method for prioritizing disease genes and inferring protein complex associations [15] and large-scale disease gene discovery by identifying human protein complexes containing known disease genes [16].

Since a first glimpse of non-random structures and dynamic behavior was observed a decade ago, a rich variety of global measures have been suggested to uncover the organizing principles behind complex networks [17]. Networked structures can emerge at different levels, from single node characteristics and the tendency of pairs of nodes to connect to each other, to the patterns exhibited by associations of three or more nodes known as motifs. These motifs are also assembled with each other defining modules and communities, which constitute an intermediate scale between single nodes and the whole networked structure.

The existence of higher order structures like modules and communities is a signature of a non-random system and provides insights into their functional organization [18]. A module represents a densely connected group of nodes that, however, is weakly connected to the remaining network. The presence of modular structure may drastically change dynamical processes that occur in networks. Spreading processes like virus epidemics and synchronization strongly depend on network modularity [19]. Moreover, modularity itself is heterogeneous and modules may have a variety of density of edges, sizes and structural features in general [20]. This large variety of features and patterns makes the detection of modularity an important albeit challenging problem.

In this work, we focus on a network that combines protein functionality information with drug interactions, in an attempt to unveil new strategies and structural features to combat complex diseases. Proteins interact with each other and define protein complexes. Protein complexes have a rich variety of functions in cells and play a key role in many human disorders. However, in spite of their importance, network studies based on protein complexes in human are still lacking. Moreover, an investigation of the interactions between all available drugs and all discovered protein complexes has not been attempted to our knowledge. The existence of modularity in this bipartite graph may lead to develop new strategies to deal with key diseases from a systemic point of view, shifting the focus from targeting individual genes or proteins to disrupting protein complexes formation. It is worth noticing, however, that several works have investigated protein complexes networks in model organisms such as yeast [21], [22]. Authors used an integrative approach in the context of gene association studies. In contrast, here we focus on human protein complex and drug associations and our methodology relies on module identification via maximization of a modularity objective function.

We used a simulated annealing algorithm to identify modules in the protein complex – drug network. The principle of this algorithm is to maximize the modularity using simulated annealing in an attempt to find low-cost configurations of structures without being trapped by high-cost local minima [23]. The algorithm mimics the cooling process of a material to improve its crystal structure. Although there have been several suggested algorithms for maximizing a modularity objective function, we selected a simulated annealing-based algorithm because it offers the highest accuracy in the detection of modularity in bipartite networks comprising up to a few thousands of nodes [24]. In very large networks, faster algorithms like greedy search, extremal optimization or spectral methods are better suited to deal with sizes of millions of nodes [18], [25]. See also the reviews [20], [26] for a detailed description of the state-of-the art in community detection. The algorithm was able to detect modules in the protein complex – drug network consisting of less than 1500 nodes and determine the global average modularity. The computation of modularity using network projections as well as the bipartite graph itself suggests that protein complexes as well as drug networks are not random networks and contain a rich and heterogeneous internal structure. The network of interactions between protein complexes and drugs reveals novel associations between key molecular compounds and diseases. The non-randomness characteristic of this network opens the possibility to explore the implications of the high modularity in the protein complex – drug space in order to unravel new pharmacological strategies. In this work, we consider the strategy of targeting a protein complex whose mutation is associated with a disease that results from a gain of function or aberrant increased activity of proteins. In the case of a disease driven by loss-of-function mutations though, additional information will need to be incorporated with the present approach in order to distinguish between increased or decreased activity.

## Methods

### Network construction

CORUM is a comprehensive database of mammalian protein complexes [27]. Entries are manually curated and annotated, including information on protein complex function, localization, subunit composition, references to Entrez gene identifiers and literature.

DrugBank is an online database of drug data, targets and action information [28]. It includes all drugs approved by the U.S. Federal Drugs Administration, as well as a large number of experimental drugs. All entries are richly annotated providing detailed information about drug chemical, pharmacological and therapeutic properties, as well as target sequences, structures and functions.

To construct a bipartite network of drugs and protein complexes, we extracted the list of protein subunits for each complex in the CORUM database, which were referenced by their SwissProt identifier. The same operation was conducted for all drug targets in the DrugBank database, resulting in a list of protein targets for each drug. The total number of drug – protein target interactions was 11950. An edge was created between a drug and a protein complex if at least one protein target of the drug was also a subunit of the protein complex. The resulting bipartite network comprised 1419 nodes (680 drugs and 739 complexes) and 3690 edges.

In all our figures colors are attributed to modules on an arbitrary basis, so that each module has a specific color. These colors are kept consistent across all figures, so that the same module appears with the same color in all figures. All network visualizations were produced using the Cytoscape software [29].

### Network projections

**Figure 1:**
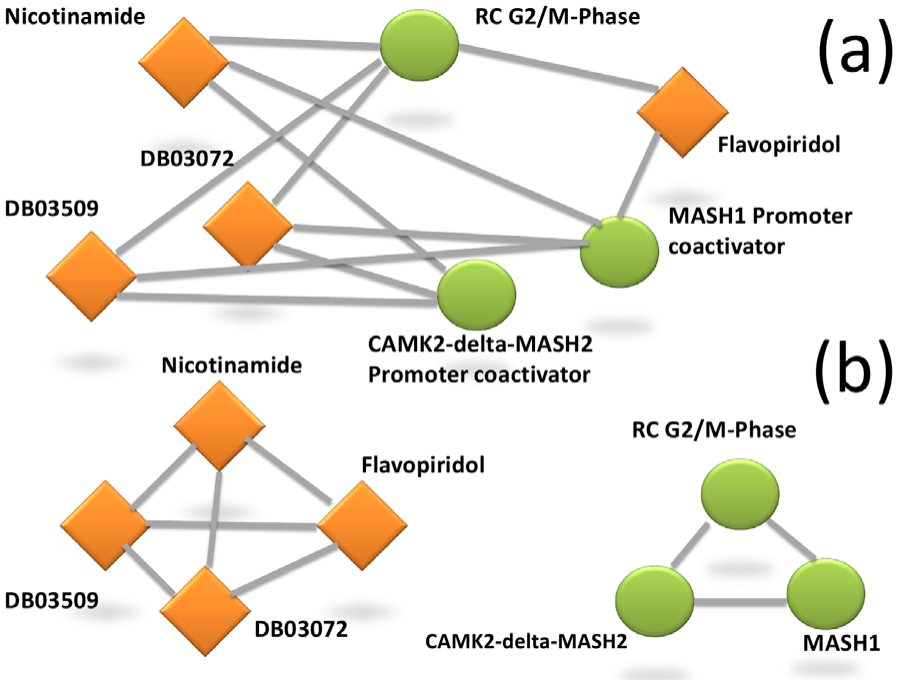
Example of bipartite and projected networks. (a) A bipartite sub-network extracted from the complex-drug network. (b) The drug and protein complex projected networks. Drugs are denoted by diamonds and complexes by circles.

Each bipartite network composed of two types of nodes can be projected (i.e. transformed) onto two networks, called projections of the original bipartite network (Figure 1). Each projected network is then composed of only one type of nodes. A bipartite graph for protein complexes and drugs can be formally defined as *G = (P, D, E),* where *P* is a set of protein complexes, *D* a set of drugs and *E* a set of edges that links two nodes from *D* and *P*. *G_p_* = (*P*, *E_p_*) represents the *P*-projection of the graph *G* in which nodes of *P* are linked together if they have at least one neighbor (*D*) in common in the graph *G*. The set of edges *E_p_* can be defined as:
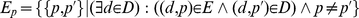
(1)The *D*-projection *G_d_* is defined dually.

### Modularity in network projections

A common characteristic of proposed algorithms to identify modularity in networks is the maximization of a modularity function. An objective function that describes modularity is usually based on the concept that the density of edges in the network is highly heterogeneous. Modules are therefore specific parts of a network where the density of edges is significantly higher than the random expectation [18], [30].
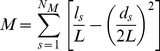
(2)In a given network with *N_M_* modules, the modularity can be computed using equation 2. In this expression, *L* indicates the number of edges in the network, *l_s_* is the number of edges between nodes in the module *s,* and *d_s_* is the sum of degrees of the nodes in module *s*. The fraction *l_s_*/*L* represents the fraction of edges within a module and (*d_s_*/2*L*)^2^ is the fraction of edges that may be inside a module by random expectation. We used a simulating annealing algorithm to find the set of modules (i.e. partition) that maximizes modularity as shown in equation 2.

The algorithm is initialized by considering that each individual node belongs to a different module (i.e. each module is composed of exactly one node). A computational temperature *T* is introduced to simulate the cooling process in materials. By starting at high temperature the systems evolves through different modularity stages overcoming local cost barriers. Maximizing modularity is equivalent to minimize a cost function defined as *C = −M.*

At each temperature the membership of nodes is randomly changed and updated according to the following probability: *p* = 1 if *C_f_*≤*C_i_* and 
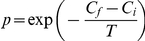
 if *C_f_*>*C_i_* where *C_i_* and *C_f_* are the costs before and after updates, respectively. The cooling down factor was set to 0.995.

### Modularity in bipartite networks

It is worth mentioning that when a projection is performed, a fraction of the information contained in the original bipartite network is lost. In order to avoid a loss of information, the modularity can be computed directly in the bipartite network itself. However, in this case an alternative definition of modularity is needed to deal with bipartite structures. As in [25], let us define nodes of type *P* (protein complexes) and nodes of type *D* (drugs) and consider a modularity functional form as follows:
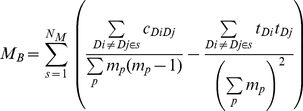
(3)where *t_Di_* indicates the total number of protein complexes a drug *D_i_* interacts with, *m_p_* indicates the number of drugs linked to the protein complex *p*, and *c_DiDj_* indicates the number of protein complexes that are simultaneously targeted by drugs *D_i_* and *D_j_*; *N_M_* is the number of modules and *s* is the module index as in equation 2. The reasoning behind equation 3 is that the average number of protein complexes in which *D_i_* and *D_j_* are expected to appear together is
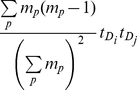
(4)Then, using this equation, we could define the bipartite modularity as the cumulative deviation from the random expectation by considering that the expected number of times a drug *D_i_* belongs to a protein complex linked to *m_p_* drugs is 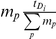.

In some cases, unweighted projections may lead to different results since, as mentioned above, much information rooted in the bipartite structure may disappear after projection [25]. The computation of modularity in the bipartite network is expected to be more accurate than in the projections. Furthermore, besides the highest accuracy of this algorithm for networks of a few thousands nodes, the method is able to identify not only an optimal attribution of the nodes into modules, but also the number of modules and their sizes. Therefore, this algorithm was selected to investigate the modularity of the protein complex-drug network (composed of less than 1500 nodes) in both the bipartite networks and their projections.

### Relationships between complexes and diseases

In order to establish connections between protein complexes and diseases, a list of Entrez gene identifiers was extracted for each complex, as downloaded from the CORUM database. The full list of genes associated to complexes was entered in the FunDO tool, which searches for associations based on the Disease Ontology [31]. This search returned a list of gene – disease associations. We conserved all associations with a Bonferroni-corrected *p*-value lower than 10^−4^. The list was made of 57 diseases, ranging from cancer (246 genes, *p* = 2×10^−109^) to Down syndrome (19 genes, *p* = 8×10^−5^). All genes were eventually re-associated to the complexes their proteins belonged to.

## Results

### Bipartite network of protein complexes and drugs

**Figure 2:**
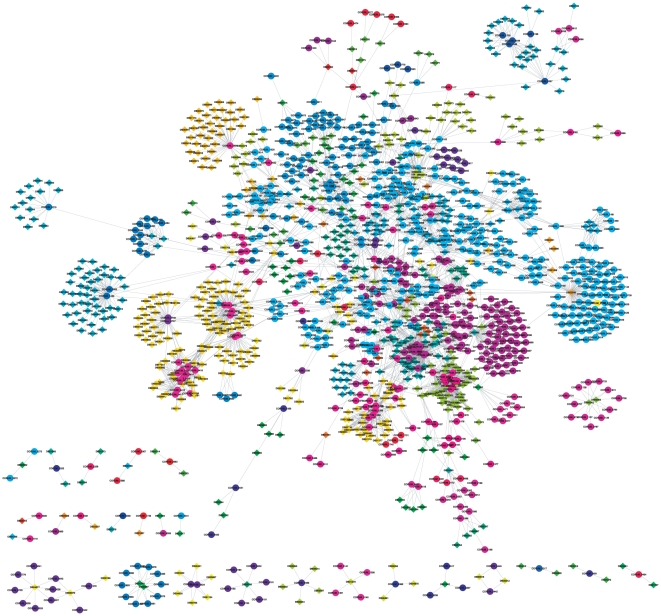
Bipartite network of protein complexes and drugs, and associated modules. A drug is connected to a protein complex if at least one protein target of the drug is also a subunit of the protein complex. Complexes are represented by circles and drugs by diamonds. Colors are attributed to modules on an arbitrary basis, so that each module has a specific color. Drugs and protein complexes are labeled by their DrugBank and CORUM identifier, respectively; mappings between these database identifiers and common names are provided in Information S2 and S3.

The bipartite network of protein complexes and drugs contains 1419 nodes (680 drugs and 739 complexes) and 3690 edges. Its structure resembles a scale-free topology, with a small number of nodes connected to many edges and the majority of nodes connected to few edges (Figure 2). Hubs can be seen among both drugs and complexes (Table 1). The most connected nodes are two drugs, flavopiridol, which is used for the treatment of leukemia, and vorinostat, which is used for the treatment of lymphoma, both interacting with over 90 complexes. A number of complexes interact with over 40 drugs, including several closely related complexes involving ER-alpha, BKCA-beta and ESR1. The degree of top drug hubs decreases faster but a number of drugs interact with 20 to 40 complexes. The network data file is provided in Information S1.

**Table 1:** Top drug and complex hubs in the bipartite protein complex – drug network.

Drug ID	Drug name	Degree	Complex ID	Complex name	Degree
DB03496	Flavopiridol	93	1004	RC during S-phase	49
DB02546	Vorinostat	91	2470	p130Cas-ER-alpha-cSrc-kinase- PI3-kinase p85-subunit	47
DB03431	Adenosine-5′-Diphosphate	44	0668	BKCA-beta2AR-AKAP79 signaling	46
DB01169	Arsenic trioxide	39	0672	BKCA-beta2AR	46
DB00054	Abciximab	30	5809	GABAA receptor	46
DB00775	Tirofiban	30	1439	PTGS2 homodimer	43
DB01254	Dasatinib	25	2657	ESR1-CDK7-CCNH-MNAT1-MTA1-HDAC2	42
DB01867	Ethylene Glycol	23	2699	ER-alpha-GRIP1-c-Jun	42
DB02010	Staurosporine	22	2700	ER-alpha-c-Jun	42
DB02116	Olomoucine	22	1003	RC	41
DB02733	Purvalanol	22	2670	ER-alpha-p53-hdm2	40
DB03428	SU9516	22	5559	CDC2-CCNA2-CDK2	40
			5862	CAV1-VDAC1-ESR1	40

### Modularity in the protein complex – drug network

Modularity is one of the emerging properties of complex networks. Modularity is not only associated to sets of nodes with specific structural functionalities but also plays a key role in the dynamic behavior of systems. Modularity is also responsible for degree correlations observed in many real-world networks.

Here, we address the problem of identifying modularity in the bipartite protein complex – drug network. The computation of modularity can be performed using either the bipartite graph itself or the projected networks. Although several works have pointed out that modularity is more reliable when computed using the bipartite approach, we computed both results for comparison. In general, it is expected that drugs will belong to the same module if they share many protein targets, regardless of whether the protein complexes themselves belong to the same module. To evaluate the statistical significance of the modularity of each network, we constructed an ensemble of randomized networks using a switching algorithm [32], [33]. This algorithm preserves the degree sequence of both drugs and protein complex nodes. It randomly selects pairs of edges, and the end points of the edges are switched preserving the degree sequence of each node.

While the drug projection with 657 nodes shows a modularity of 0.7755 (0.1459±0.0010), the protein complex network with 723 nodes has a modularity of 0.6500 (0.1191±0.0016). Values of the modularity and standard deviations for a trial of 20 randomized networks with the same degree sequences as the original networks are shown in parentheses. These values show that the analyzed networks have a significantly higher modularity than expected by chance. The number of modules in the drug projection is 23, while 17 modules were detected in the protein complexes projection. It is worth noticing that in spite of the increasing density of edges in the projected networks, the modularity is still much higher than what we could expect in a random network. The values of modularity in the randomized networks are still around six times lower than in the real projected networks, giving high statistical significance to the result in projections (*p*<10^−30^).

Network analysis shows that the drug projection is characterized by a high mean degree <*k*> = 15.48 and a diameter *d* = 7. It also shows a small average shortest path length <*l*> = 2.8 and a high mean clustering coefficient *C* = 0.84 compatible with a small-world network. Analysis of the protein complex projection leads to similar values, with a slightly higher mean degree <*k*> = 20.7 and diameter *d* = 8. Similar values are found for average shortest path <*l*> = 2.34 and clustering degree *C* = 0.84.

Visualizations of these projections with the identified modules are shown in Figures S1 and S2. Moreover, we were able to compute the modularity using the original bipartite graph. Both projections are not extremely dense, therefore it is expected that, in this case, both approaches should lead to similar results for the average modularity. However, memberships of nodes as well as the number of modules may differ. As general rule, when projections are too dense, the computation using bipartite graphs is preferable. The results computed in the bipartite network show a modularity of 0.8244 and 0.7615 for drugs and protein complexes, respectively. We obtained 48 modules of drugs and 42 modules of complexes in the bipartite network. As expected, the modularity values show a good correlation between both approaches. Figure 2 shows the modules identified in the bipartite network (mappings between database identifiers and names of drugs and complexes are provided in Information S2 and S3). The modularity analysis reveals that these networks are strongly different from random networks and are characterized by a highly modular structure. It is worth noticing that although the overall modularity in projected and bipartite networks are very close, around 0.75 in average, a larger number of modules are isolated by computation in the bipartite graph. This is in part a consequence of the smaller number of edges in the original bipartite graph, which tends to increase the accuracy of module detection. For example, a large module can potentially be more precisely identified as two weakly connected modules. Furthermore, there is a large number of modules composed of a single node.

### Hierarchy and centrality measures

**Figure 3:**
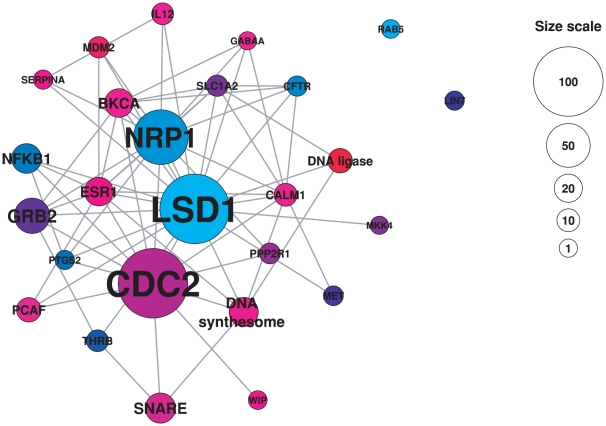
Projected network of complex modules. Each module of the protein complex – drug bipartite network was shrunk into a node and the complex projection of the resulting network is represented. Modules are named according to a representative complex hub inside the module; only names of large modules are displayed for clarity. Colors are attributed to modules on an arbitrary basis, so that each module has a specific color. The size of nodes is proportional to the number of complexes in each module; a size scale is displayed on the right-hand side of the figure. To assign names to condensed nodes, we chose a representative member of each module by selecting the drug with the highest degree inside the module.

**Figure 4:**
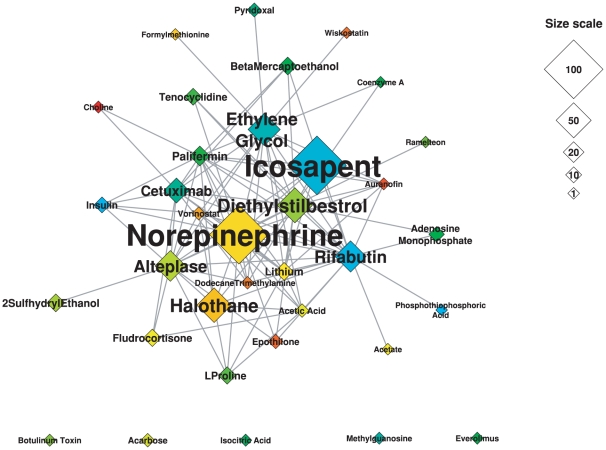
Projected network of drug modules. Each module of the protein complex – drug bipartite network was shrunk into a node and the drug projection of the resulting network is represented. Modules are named according to a representative drug hub inside the module. Colors are attributed to modules on an arbitrary basis, so that each module has a specific color. The size of nodes is proportional to the number of drugs in each module; a size scale is displayed on the right-hand side of the figure. To assign names to condensed nodes, we chose a representative member of each module by selecting the complex with the highest degree inside the module. In the case of protein complex names formed by association of numerous protein names, we selected the protein occurring most frequently in complexes connected to the complex of highest degree.

**Figure 5:**
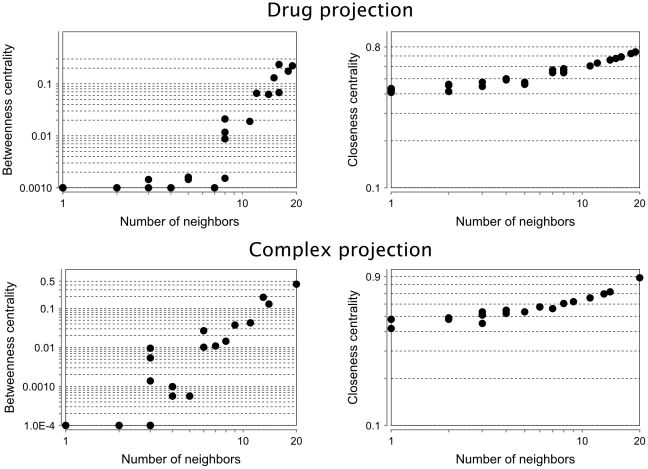
Network metrics in projected networks of modules. Top panels are from the drug projection and bottom panels from the complex projection. Left side panels represent betweenness centrality and right side panels closeness centrality.

In order to get a clearer view of the identified modularity structure, we can shrink all nodes that belong to the same module into one node, with its size proportionate to the number of members in the module (Figures 3 and 4). This transformation represents a projection of the modules into a higher layer, simplifies the structure and allows us to obtain a global view of its hierarchy [34]. A network analysis based on centrality measures of both complex and drug projections reveals a correlation with node degree highlighting the non-random nature of the modularity observed in our analysis (Figure 5). Here, we examine the betweenness centrality (*B_i_*) that characterizes a network beyond local information and reflects the role played by a node in the global network architecture. It is calculated as the fraction of shortest paths between node pairs that pass through a given node. In contrast, the closeness centrality (*C_i_*) measures how close a node *i* is to all others in the same network and is defined as the average mean path between a node *i* and all other nodes reachable from it.

The distribution of betweenness centralities reveals distinctions between a few modules occupying highly central positions and a majority of more peripheral modules. Among modules of complexes, the LSD1 module has a strikingly high betweenness centrality (0.42) compared to other modules. This complex contains hubs such as the LSD1 complex, the Kaiso-NCOR complex and the HCF-1 complex, which have major functions in controlling the cell cycle and DNA processing. Differences are less marked among modules of drugs, but high betweenness centralities are exhibited by the ethylene glycol module (0.24) and rifabutin module (0.22). The distribution of closeness centralities is more even but again the LSD1 module exhibits a privileged central position with a closeness centrality of 0.88. These high centrality values suggest the existence of bridging nodes, a non-random feature of networks. Recently, this feature was considered in detail in [35] showing that bridging nodes may play a crucial role in network regulation. Similarly, links that bridge modules could also be examined in detail in combination with gene expression profiles, offering a new way to exploit the intrinsic modularity and topological features of the drug – protein complex network.

### Linking protein complex modules to protein-protein interactions

**Figure 6:**
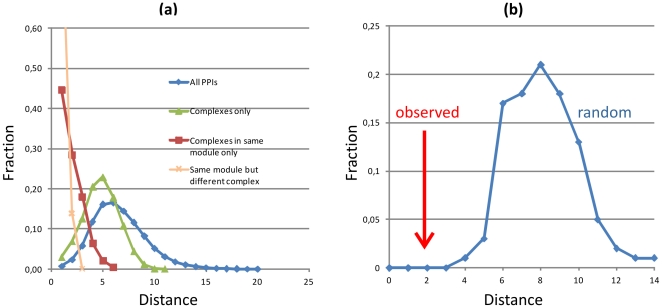
(a) Distribution of shortest distances in the entire protein-protein interaction network (blue curve) and in interactions between all proteins involved in complexes (green curve); interactions between proteins involved in complexes and belonging to the same module are shown by the red curve, and these belonging to the same module but not to the same protein complex are shown by the orange curve. (b) Observed characteristic path length (red arrow) and distribution of characteristic path lengths for the random control (blue curve). We generated 100 independent samples by randomly shuffling protein associations while keeping each node degree unchanged.

In previous sections, we have constructed a set of functional modules of protein complexes based on identified associations with drugs. Since proteins linked by drug associations should in principle be involved in related disorders, the proteins within such modules are expected to interact preferably with one another than with other proteins. To test this hypothesis, we analyzed the relationship between modules of protein complexes and a generic protein-protein interaction (PPI) network. The core dataset of human protein-protein interactions from the Database of Interacting Proteins (DIP, version 2010-10-10) [36] was used as a reference. The computation of the shortest path lengths between proteins is a measure of the proximity of proteins in a PPI [37]; we thus use this network metric to evaluate whether the identified modules of protein complexes have a biological interpretation. If the proteins that belong to identified modules appear in highly interlinked local regions of the PPI network, the average shortest paths between these proteins would be smaller than the average shortest path between other proteins in the PPI. In Figure 6a, the blue curve shows the distribution of shortest paths between proteins in the entire PPI network; the green curve shows the distribution of shortest paths between the subset of proteins involved in complex formation, which is only slightly shifted towards smaller weights compared to the original distribution; the red curve shows the distribution of shortest paths between proteins involved in complex formation and belonging to the same protein complex module, and the orange curve shows the subset of these proteins belonging to the same module but not to the same protein complex. The comparison shows a significant shift of protein-protein pairs belonging to the same module (red curve) towards smaller weights (*p* = 0.001); almost half of these proteins are adjacent in the PPI. The observation that protein-protein pairs that are in the same module but not in the same protein complex are also shifted toward smaller weights (*p* = 0.001) indicates that the shift is not only due to protein pairs within the same complex, but also to proteins connected through other forms of interactions. In Figure 6b, we furthermore compare the average shortest path in the latter network to a random control of 100 networks of the same size preserving the degree distribution; the observed characteristic shortest path between proteins belonging to the same module (red arrow, 1.942) is significantly smaller than the expected value for the random control (*p* = 0.003). These findings highlight the biological significance of modules identified in the protein complex – drug network.

### Relationships to diseases

Since the modularity analysis in the protein complex – drug bipartite network revealed a high modularity and a striking non-randomness, these modules are likely to be related to common factors, in this case common diseases. To unravel the relationship between such modules and diseases, we investigate the origin of this modularity in two particular examples. It is important to note that our approach is targeted towards gain-of-function mutations, where the disease results from aberrant increased activity of proteins.

#### Example 1: Leigh disease

Leigh disease is an inherited neurometabolic disorder that affects the central nervous system, causing degradation of motor skills and eventually death. The disease has been linked to mutations in mitochondrial DNA, affecting energy production and causing a chronic lack of energy in cells [38].

**Figure 7:**
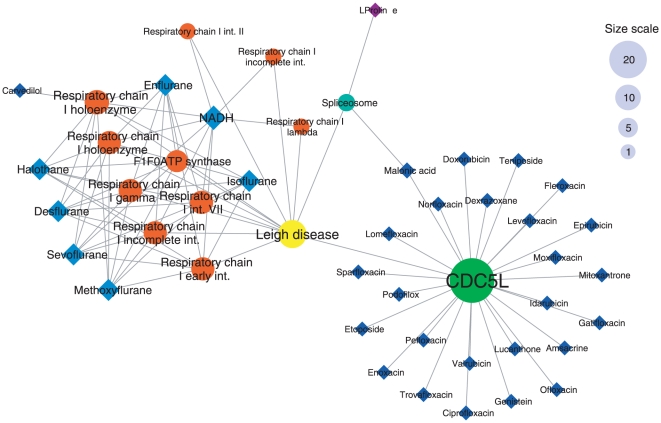
Tripartite network of drugs and protein complexes connected to Leigh disease. Links between the disease node and protein complexes represent associations between genes involved in these complexes and the named disease, as specified by the Disease Ontology. Links between protein complexes and drugs are the same as in our bipartite network, meaning that a drug is connected to a protein complex if at least one protein target of the drug is also a subunit of the protein complex. Complexes are represented by circles and drugs by diamonds. Colors are attributed to modules on an arbitrary basis, so that each module has a specific color. The disease node is represented by a yellow circle. The size of nodes is proportional to the degree of each node; a size scale is displayed on the right-hand side of the figure.

The network of protein complexes connected to Leigh disease reveals two major and clearly distinct components (Figure 7). On the one side, a module of highly related and interconnected protein complexes related to the respiratory chain I, connected to anaesthetic drugs such as fluranes and halothane. On the other side, the large CDC5L complex, formed by the assembly of 30 proteins [39]. A large number of drugs are associated to this complex, however the association between CDC5L and Leigh disease has not been mentioned so far. A multitherapeutic strategy involving both targeting respiratory chain complex I and CDC5L formations could thus be envisaged for a more comprehensive targeting of the factors associated to Leigh disease.

#### Example 2: Parkinson disease

Parkinson disease is one of the biggest health issues facing many nations with an ageing population. It is a degenerative disorder of the central nervous system that impairs several motor-related functions and cognitive processes. There is no known cure for the disease, but several drugs are used to provide relief from the symptoms. Parkinson disease is a typical example of a complex disease, whose causes are multifactorial and whose treatment requires new polypharmacological approaches [40]. Mutations of specific protein complexes have been linked to Parkinson disease [41].

**Figure 8:**
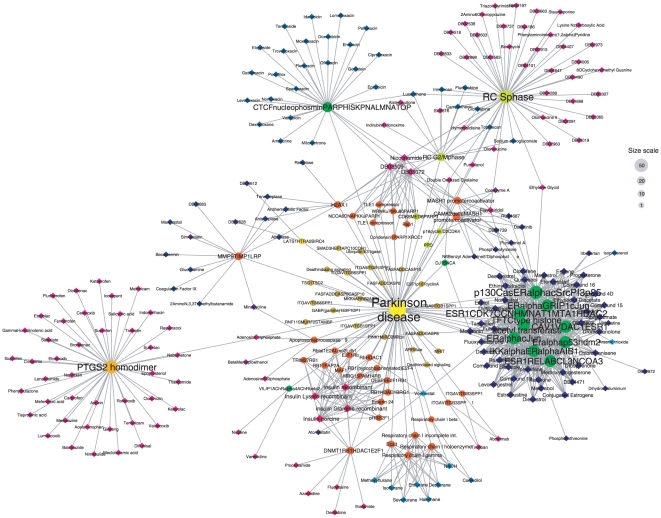
Tripartite network of drugs and complexes connected to Parkinson disease. Links between the disease node and protein complexes represent associations between genes involved in these complexes and the named disease, as specified by the Disease Ontology. Links between protein complexes and drugs are the same as in our bipartite network, meaning that a drug is connected to a protein complex if at least one protein target of the drug is also a subunit of the protein complex. Complexes are represented by circles and drugs by diamonds. Colors are attributed to modules on an arbitrary basis, so that each module has a specific color. The disease node is represented by a yellow circle. The size of nodes is proportional to the degree of each node; a size scale is displayed on the right-hand side of the figure.

Interestingly, the network of protein complexes and drugs connected to Parkinson disease (Figure 8) presents similar characteristics as the Leigh disease network, albeit on a larger scale. On the one side, a module of strongly interconnected complexes can be observed, which are mainly linked to ESR1 and ER-alpha. These complexes are connected to a module of about 40 drugs, including for example desogestrel, progesterone and letrozole. On the other side, a few isolated complexes are connected to the disease, which are themselves targeted by a large number of drugs; these are principally the RC S-phase, RC G2/M phase, PTGS2 homodimer, CTCF-nucleophosmin-PARP-HIS-KPNA-LMNA-TOP and MMP-9-TIMP-1-LRP complexes. In addition, a group of 21 complexes are connected to the disease, which are not targeted by any drug; this group includes for example the ITGAV-ITGB1-SPP1 and the TSC1–TSC2 complexes. In this example, the network highlights a certain bias of current pharmacological approaches, which tend to focus on a few targets for which multiple drugs are developed, while on the other side other potential components involved in the disease are not targeted. More comprehensive treatments of complex diseases such as Parkinson may require more systematic approaches attempting to target all the factors contributing to the disease.

For example, flavopiridol is seen to be connected to five different complexes associated to Parkinson disease, which are the RC S-phase, RC G2/M-phase, p16-cyclin D2-CDK4, CDK8-MED6-PARP1 and ESR1-CDK7-CCNH-MNAT1-MTA1-HDAC2 complexes (Figure 8). Interestingly, the potential role of flavopiridol in inhibiting cyclin-dependent kinase cdk5, which is inappropriately elevated by neurodegenerative conditions, has already been suggested [42] but the drug is not currently used in this context.

Vorinostat, constitutes a module on its own in this network with high betweenness centrality, highlighting its unique position as an interactor between complexes involved in different cellular functions, which are the DNMT1-RB1-HDAC1-E2F1, ESR1-CDK7-CCNH-MNAT1-MTA1-HDAC2, RB1-HDAC1-BRG1 and Rb-HDAC1 complexes. So far none of these complexes was associated to Parkinson disease; however aberrant activation of the RB1-E2F pathway was observed to mediate neuronal cell death and its inhibition was proposed as a possible strategy for neuroprotection [43]. These examples show how a network integration of heterogeneous datasets can highlight drugs with important polypharmacological properties and offer new insights into possible ways to combat complex diseases.

## Discussion

In this work, we have considered both network projections and the bipartite graph structure to investigate the modularity of the complex – drug interacting space. Network projection is a useful technique in graph theory that allows the transformation of bipartite networks into unipartite graphs, where standard network metrics can be easily applied. However, since real-world data are rich in multipartite relationships, it is desirable to compute network metrics such as modularity in the original graph. In addition to the loss of information when a projection is performed, each protein complex of degree *k* in the bipartite graph generates *k*(*k*−1)/2 edges in the drug projection in our network, which leads to a sharp increase in edge density in the projected network [44]. This does not only makes the computation of modularity less accurate but also drastically increases the computational time. Our approach has been successful in considering modularity derived from the bipartite structure.

Network analysis has provided insights into the non-random nature of the system under study. The fact that the drug-protein complex network is non-random has allowed us to identify modules that can be associated to particular diseases. In a random network, the finding of modules would not have any significance, since each node carries almost the same topological information. The existence of modularity implies that the network has intrinsic features that could be exploited in future molecular therapies. The finding that the drug – protein complex network exhibits non-random metrics and high modularity opens a therapeutic option when the system is linked to particular diseases.

Several recent works have analyzed the topology of a network as a preliminary stage to consider possible medical applications. In [45], the modularity in protein interaction networks was linked to the prediction of breast cancer outcome. They combined topological analysis with gene expression data and examined the difference in modularity in two breast cancer patients. As a result, this work encouraged the search for multi-modal therapies targeting hubs in the network that displayed altered modularity in disease. This example illustrates the importance of having non-random structures in the current human interactome. In our study, we could potentially merge our networked structure with gene expression profiles to detect co-expression of hub partners and perform similar analysis for several disease stages or disorders. Additional examples of the utility of network analysis for therapeutic approaches can be found in [46]. In general, molecules involved in a specific biochemical process or disease may have similar neighbors that also participate in the same or closely related pathways or disorders. A well-defined neighborhood of the interactome is then referred as a “disease module”. In [12], Goh et al., identified a high number of physical interactions between the products of genes associated with the same disorder, representing a tenfold increase relative to random expectation. Similar observations were made in [47], [48]. Analogous comparisons to random expectation were done in our study with drug – complex networks. Furthermore, in our case a probabilistic algorithm that maximizes the modularity of the whole network identified each module as well as the global network modularity.

Identification of communities and modules is computationally challenging and represents a central issue in network science [26]. The problem is that there are several possible definitions of modularity and there is not yet an agreement on which one may lead to a better representation of the same phenomena. This is particularly true in the case of bipartite and directed networks. One of the issues of modularity detection is the overlapping. The existing deterministic and stochastic methods used for large networks are able to find separated communities, whereas several of the actual networks are made of overlapping cohesive groups of nodes [26]. Several algorithms like CFinder [49] and ModuLand [50] have recently been proposed to identity modules with overlapping structure. This problem affects the nodes located at the boundary of modules, and a node may then belong to several modules. Farkas et al. also showed that bridging nodes may play a crucial role in network regulation [35]. In our work, we have focused on a bipartite network and computed the modularity in their projected networks but also in the bipartite network itself. For technical reasons, like the growing density of nodes in the projections, the computation of network metrics in the bipartite network is usually preferable [25]. Here, we have used a simulated annealing algorithm that detects modules with high accuracy in networks comprising up to a few thousands of nodes according to computational experiments done in [24]. Furthermore, there is no version of the CFinder algorithm to detect modularity with overlapping in bipartite networks. Therefore, we did not use CFinder for our analysis. In overlapping algorithms, some nodes may be assigned to different or several modules simultaneously. Moreover, another issue is that there is still no consensus about a quantitative definition of the concept of overlapping community, and most definitions depend on the method adopted [26]. This question could certainly be the object of further study together with the identification of bridging nodes and links between the determined modules. We could then examine whether the two end-nodes of these bridging links may have any biological importance in a similar way as done in [35].

Connections between protein complexes and diseases have been suggested for decades [51], and several recent studies have linked the formation of specific protein complexes to human disorders. The Crumbs complex participates in various human diseases, including blindness and tumour formation [52]. Alzheimer's disease is characterized by amyloid plaques, which are built by a high molecular weight protein complex containing presenilin (PS), nicastrin, Aph-1 and Pen-2 [53]; presenilins are thought to be important drug targets for this disorder [54]. Mutations in genes encoding structural subunits of complex I, a mitochondrial complex involved in energy production in the form of ATP through the process of oxidative phosphorylation, have been identified as a cause of devastating neurodegenerative disorders with onset in early childhood [55]. The IKK complex is an essential regulator of NF-kappa-B activation, which is a major regulator of the defense against pathogens, antigen-specific adaptive immune responses or chemical stress. Dysregulated NF-kappaB signaling was linked with the onset or progression of various diseases, including cancer, chronic inflammation, cardiovascular disorders and neurodegenerative diseases [56]. Protein-protein interactions and complex formation were also named as highly promising drug targets for trypanosome induced diseases, due to the low sequence identities between some parasite proteins and human ones [57].

For those reasons, protein complexes are increasingly considered as potential targets for novel therapies to treat complex diseases. In opposition to the predominant single drug – single target – single disease paradigm, complexes necessarily involve multiple proteins, thus multiplying the possibilities for disrupting their formation. There are already several successful examples of inhibitors of protein-protein interactions and this approach is considered with interest by the pharmaceutical industry [58]. Network-based approaches can help to identify promising targets since they enable us to consider the complete set of relationships between protein complexes and related diseases. One of their advantages is to reveal associations across heterogeneous datasets; for example we have shown that vorinostat has multiple associations with Parkinson disease which were not previously reported although individual associations between the drug's targets and neuronal cell death were known. The integration of heterogeneous datasets can highlight drugs with important polypharmacological properties and thus offer new insights into possible ways to combat complex diseases. We also highlighted the non-randomness and high modularity of this network, and described how such modules are linked to diseases. We observed that protein pairs belonging to the same module tend to be more closely connected through general protein-protein interactions. Additional assessment of the biological significance of modules could be obtained by integrating other types of high-throughput datasets to the network, such as gene expression data.

The distinction between types of diseases driven by gain- or loss-of-function mutation will influence drug development strategies. In our approach, we consider the strategy of targeting a protein complex whose mutation is associated with a disease that results from a gain of function or aberrant increased activity of proteins. In the case of a disease driven by loss-of-function mutations though, additional information will need to be incorporated with the present approach in order to distinguish between increased or decreased activity. The application of treatment strategies without considering this distinction could cause further inhibition of function of an affected complex that already has a deleterious effect. We here illustrated our strategy by the examples of Leigh and Parkinson diseases. In the case of Leigh disease, there is currently no known drug to treat that disease, treatments aim at alleviating its symptoms mainly by providing thiamine or other vitamins to stimulate mitochondrial metabolism. There is currently no cure for Parkinson disease either, in the sense that drugs cannot eliminate the disease but mainly slow its progression and relieve its symptoms. Most existing anti-Parkinson drugs act as neuroprotectants by increasing the level of dopamine or reducing the level of acetylcholine [59]. While our approach can be extended to other diseases, it is worth noting that targeting functional protein complexes is only one potential aspect in the design of therapies for complex diseases. In many cases, diseases result from non-functional proteins, and drugs are needed that alleviate those symptoms and not drugs that directly target those affected proteins. Developing new therapeutic strategies involves taking these multiple aspects into account, which is where systems biology and tools of network medicine [46] can play a role by providing novel approaches to integrate these vast amounts of heterogeneous information, thereby enabling a more global interpretation.

## Supporting Information

Information S1 **Data of the drug – complex bipartite network.** This file can be readily imported into the Cytoscape software for visualization by using the “Import Network from Table” command. Each row in the data file represents a connection between a drug and a complex. Column 1 contains DrugBank drug identifiers, column 2 contains CORUM complex identifiers.
(TXT)

Information S2 **Mapping between DrugBank identifiers and common names of drugs.**
(XLS)

Information S3 **Mapping between CORUM identifiers and common names of protein complexes.**
(XLS)

Figure S1 **Projection of the drug – complex bipartite network into the space of protein complexes.** Complexes are labeled by their CORUM identifier; the mapping between database identifiers and common names of complexes is provided in Information S3.
(PDF)

Figure S2 **Projection of the drug – complex bipartite network into the space of drugs.** Drugs are labeled by their DrugBank identifier; the mapping between database identifiers and common names of drugs is provided in Information S2.
(PDF)
