# Supplementary figures and images for: Modularity in Protein Complex and Drug Interactions Reveals New Polypharmacological Properties
Source: PLoS One. 2012 Jan 18;7(1):e30028. doi: 10.1371/journal.pone.0030028 (PMC3261189; doi:10.1371/journal.pone.0030028)

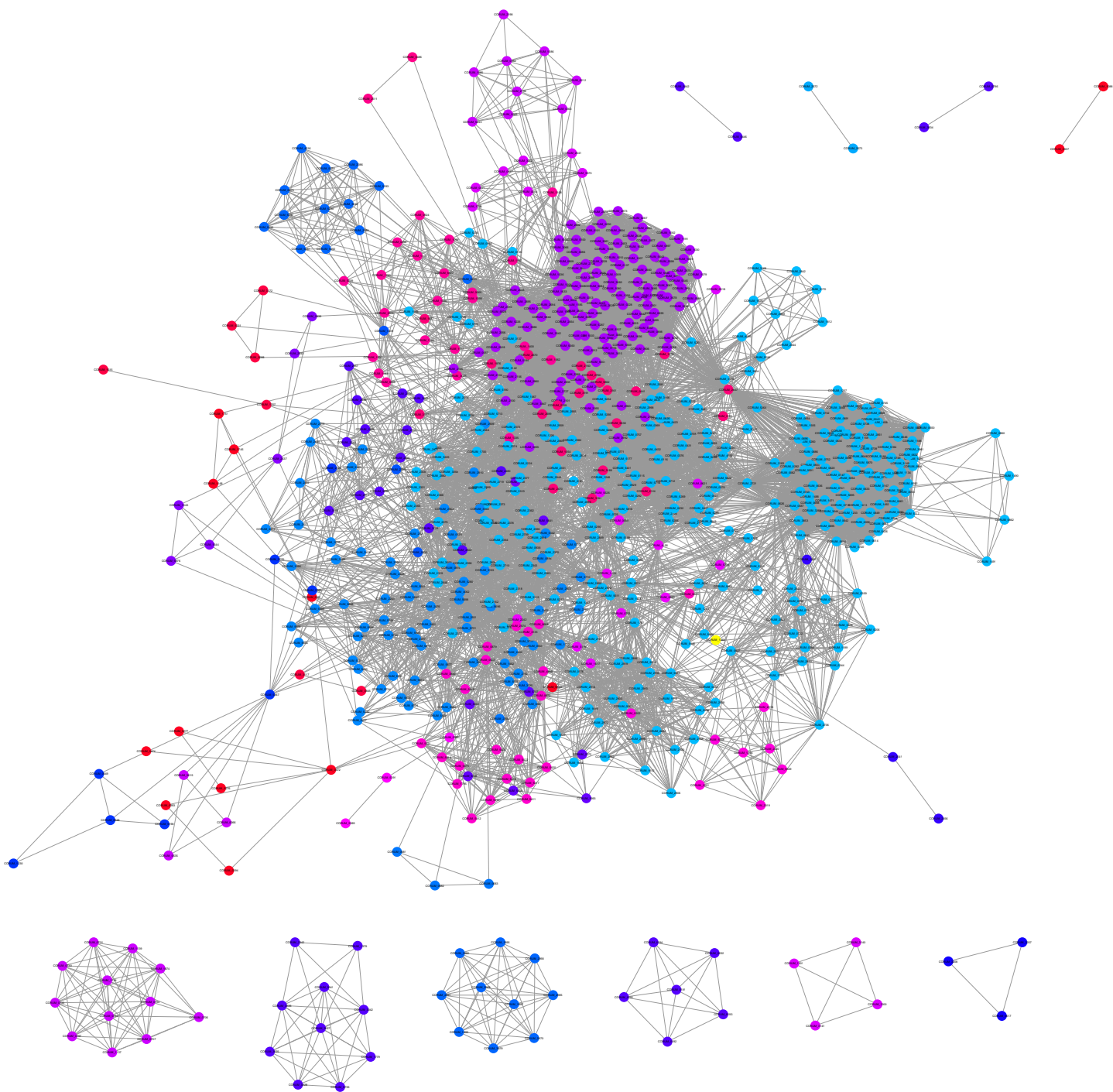

Supplement: Figure S1 — Projection of the drug – complex bipartite network into the space of protein complexes. Complexes are labeled by their CORUM identifier; the mapping between database identifiers and common names of complexes is provided in Information S3. (PDF) [file pone.0030028.s004.pdf]

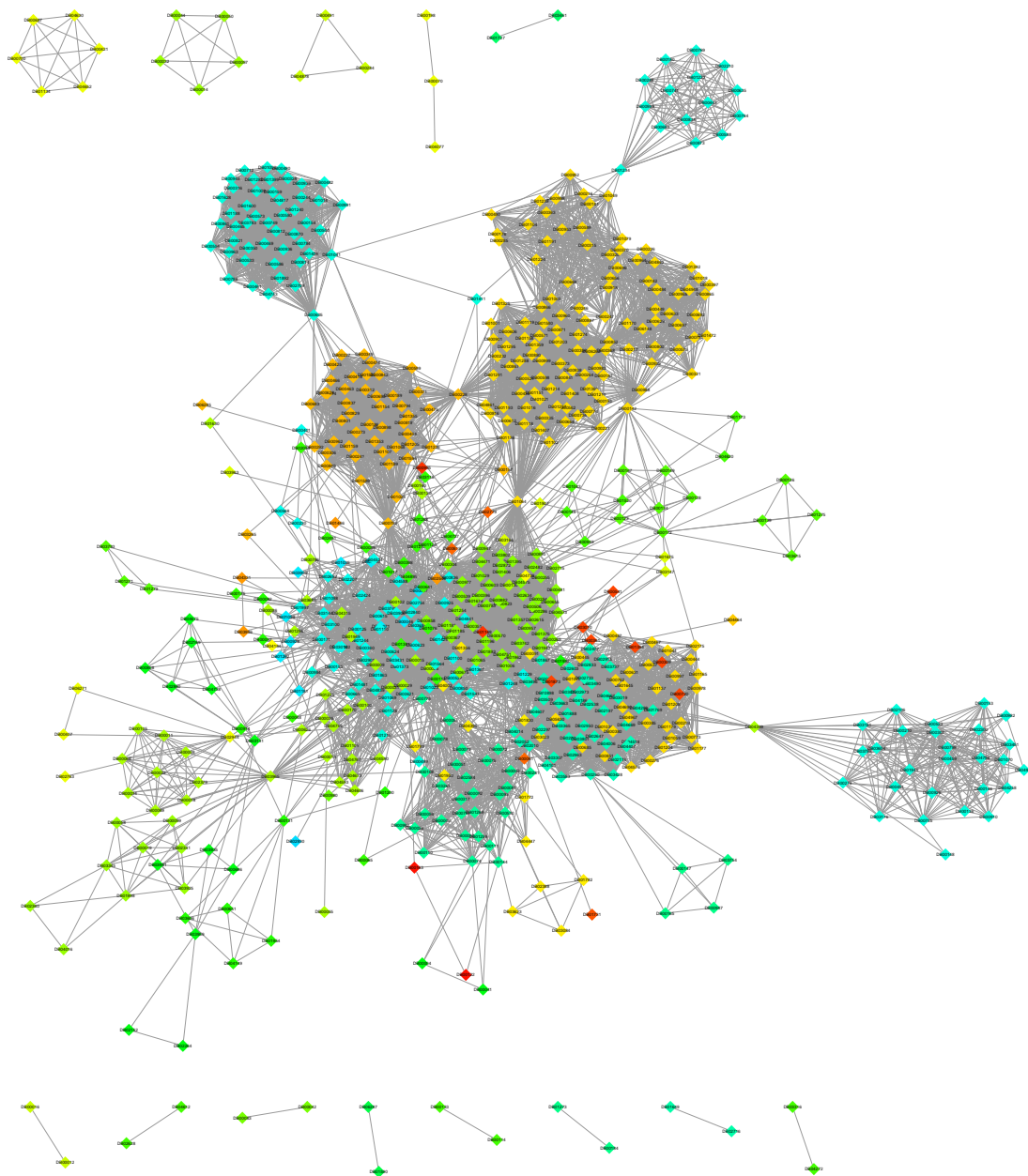

Supplement: Figure S2 — Projection of the drug – complex bipartite network into the space of drugs. Drugs are labeled by their DrugBank identifier; the mapping between database identifiers and common names of drugs is provided in Information S2. (PDF) [file pone.0030028.s005.pdf]
